# How Can Communication about Plant-based Foods Support Sustainable Food System Transformation? Nine Recommendations for Government, Industry and Citizens

**DOI:** 10.1007/s40572-025-00489-4

**Published:** 2025-06-28

**Authors:** Tess Davis, Cristina Stewart, Esther K Papies

**Affiliations:** 1 MRC/CSO Social and Public Health Sciences Unit, School of Health and Wellbeing, University of Glasgow, 90 Byres Road, Glasgow, G12 8TB UK; 2 Behavioural Science Institute, Faculty of Social Sciences, Radboud Universiteit, Nijmegen, Netherlands

**Keywords:** Food Communication, Plant-based Foods, Sustainable Eating Behaviour

## Abstract

**Purpose of Review:**

Immediate and widespread action is necessary to minimise the harmful consequences of the current food system. Mainstream omnivore diets contribute significantly to greenhouse gas emissions, environmental degradation and biodiversity loss. Therefore, switching to a more sustainable, plant-based diet is necessary for reducing the adverse effects of the current food system. Communication can play a key role in transitions towards sustainable eating behaviour. This paper will explore how communication about plant-based foods can be used most effectively across three sources: (1) government, (2) food industry and (3) citizens.

**Recent Findings:**

Currently, the food industry drives the majority of communication about plant-based foods. Governments seldom communicate about plant-based foods in consumer-facing guidelines, with non-governmental and intergovernmental organisations instead filling the gap. Citizens are then exposed to, and seem to internalise, commercial communication about plant-based foods. This communication favours traditional, meat-centric norms and positions plant-based foods as an unenjoyable, inaccessible and expensive alternative to meat and dairy products.

**Summary:**

We present 9 recommendations to improve communication about plant-based foods to support more widespread adoption of sustainable diets. These recommendations are tailored to different sources of communication but centre around three main ideals, namely to make communication about plant-based foods (1) consistent, (2) reward-focused and (3) practical. These recommendations can help to tackle misconceptions about plant-based foods and encourage the widespread adoption of sustainable diets that is necessary for human and planetary health.

## Introduction

*‘Eat food. Not too much. Mostly plants.’* - Michael Pollan.

The climate crisis is not a ‘far-in-the-future’ theoretical scenario [1] but a catastrophic, irreversible and escalating existential threat to human and planetary health [2]. As such, tackling high-impact consumer behaviours is crucial for mitigating the anthropogenic drivers of climate change [3, 4]. Livestock production has the greatest impact on global GHG emissions, i.e. 12–18%, in the food sector [5–9], and is the leading driver of environmental degradation, deforestation and biodiversity loss [10–13]. Without substantial reduction in meat and dairy consumption, particularly in industrialised nations, the environmental impact of the food system could increase by 50–90% by 2050 [14].

Recent research has shown that, in the UK, plant-based diets (which we refer to here as excluding the consumption of all animal products; [15] typically have less than half the environmental impact of high-meat diets, while low-meat diets have around a 30% lower impact than high-meat diets [16]. Similarly, the EAT-Lancet Commission [17] developed an optimal diet for human and planetary health, which recommends that per day, citizens should aim to consume over 1000 kcals of fruits, vegetables, nuts and legumes (43% of daily intake), and consume less than 150 kcals of meat and fish (5% of daily intake), and around 175 kcals of dairy and eggs (7% of daily intake). For comparison, current animal product consumption accounts for 30% of total caloric intake in the EU [6].

Nevertheless, people are hesitant to reduce their animal product intake. For example, omnivores seldom trial meat free days, replace meat or dairy with plant-based proteins, or choose fully plant-based dishes [18–21]. In the UK, consumer dietary trends show small shifts in animal product consumption in recent years, with the National Diet and Nutrition Survey reporting a 17% reduction in meat intake between 2008 and 2019 [22]. Notably, although global beef consumption needs to decrease by 89% to stay within planetary boundaries [14] only a 30% reduction was observed on average, which was coupled with an increase in white meat consumption [22]. Furthermore, 34% of participants exceeded the daily recommended intake of red and processed meat [22].

This paper examines the current and potential roles of communication in promoting a shift away from meat and dairy consumption and towards more plant-based foods. Here, we define plant-based foods as those which do not include any meat, fish or dairy products, or any other products derived of animal origin [23]. This includes a wide diversity of unprocessed, processed and ultra processed plant-based foodstuffs, including fruits, vegetables, pulses, legumes, wholegrains, nuts, ultra processed meat and dairy alternatives, tofu, seitan and tempeh [see: 24]. Additionally, we define communication as how people create meaning psychologically, socially and culturally [25, 26]. In the context of the food system, this can include explicit forms, such as dietary guidelines, advertising, and social media discourse, as well as implicit forms, such as product placement, choice architecture and social modelling. These communications are shaped by aspects such as market pressures, agriculture subsidies, and actor values, which in turn shape cultural norms, attitudes, and psychological representations of foods [27–29].

Communicating effectively about the benefits of plant-based foods specifically may be an important factor in supporting mainstream transitions toward more sustainable eating behaviour [30]. In this literature review, we discuss communication from three sources that each play a key role in maintaining and transforming the food system: (1) government, (2) food industry, and (3) citizens [see: 31]. For each, we will first discuss how communication may affect attitudes and behaviour toward plant-based foods and then recommend methods to improve communication across sectors to support sustainable food system transformation. This review predominantly draws on research from the UK, Western Europe and North America, due to the concentration of research and evidence surrounding plant-based food communications from these high-income nations, but more importantly, as reducing the overconsumption of meat and dairy in these populations is particularly urgent for global health and environmental sustainability [32]. Nonetheless, the principles discussed here may apply to other nations and populations with high meat consumption.

## Government Communication about Plant-based Foods

### Current Communication: Governments Seldom Communicate about Plant-based Foods

Despite the significant food system transformations needed, analogous with general net zero targets, Paris Agreement goals and public health priorities (see: 12), few governments communicate directly about plant-based foods. A recent study on global food-based dietary guidelines, i.e. the main output in which governments communicate about food to citizens, found that only 6 out of 58 countries (i.e. Belgium, Denmark, Estonia, France, Poland, Sweden) included sustainability-related recommendations to reduce meat intake, and only 2 countries (i.e. Estonia, Sweden) included recommendations to reduce dairy intake [33]. These public-facing recommendations varied widely in terms of information about the negative environmental impact of animal products, supporting evidence and action points for dietary change. As a result, communications about plant-based foods are missing from public-facing dietary policy documentation in most countries.

Other methods of communicating about sustainable food consumption, such as environmental impact labelling requirements on food packaging, are more popular among policymakers. These ‘eco-labels’ or ‘carbon labels’ also receive public support as a non-intrusive intervention [34] and are set to be implemented in Europe and the UK (see: 36). However, ecolabels have chiefly been tested in hypothetical experimental conditions [36–38]. Their impact in real-life contexts is minimal [39], potentially due to lack of familiarity, inconsistency of ecolabel types across different products, and the cognitive burden among citizens in understanding and quantifying what these labels mean [35, 40, 41]. As such, top-down communication from governments needs to go beyond just providing information to influence consumer dietary behaviour.

### Contextualising Communication: NGOs and IGOs Fill the Gap of Top-Down Communications about Plant-based Foods

In the absence of policy communications about plant-based foods, non-governmental organisations (NGOs) and intergovernmental organisations (IGOs) such as the WWF, IPCC and the UK Climate Change Committee have published a range of independent reviews, reports and resources that emphasise the urgent need to shift towards more sustainable, plant-based diets [42–44]. In the UK, these include the National Food Strategy [45], Better by Half Roadmap [46] and a recent report on the environmental and health benefits of plant-based options [47]. Not-for-profit organisations have also released a number of publications promoting plant-based consumption, such as the Good Food Institute’s State of Global Policy report [48], and the German Nutrition Society’s Eat and Drink Well recommendations [49]. These outputs also tackle common perceived barriers relating to sustainable diets, such as protein adequacy concerns for plant-based foods in industrialised countries where there is no protein deficiency issue at a population level [50]. Although these publications are important, there is now a reliance on communications from NGOs and IGOs for evidence, recommendations and guidance surrounding plant-based foods, which signals a lack of support for these messages from governments and public health institutions themselves. Moreover, these reports, including the National Food Strategy, which was commissioned specifically for the UK government, has not been integrated into food-related policies or actioned to develop effective top-down communication strategies for sustainable food consumption.

The absence of government-regulated communications about plant-based foods can also lead to pro-meat or pro-dairy messaging from organisations with strong links to industrial livestock farming. For example, the 2024 ‘Let’s Eat Balanced’ marketing campaign from the Agriculture and Horticulture Development Board, which is sponsored by the Department for Environment, Food and Rural Affairs [51], promoted the nutritional benefits of red meat and dairy ‘for a healthy and sustainable diet’ in a range of social media and TV adverts across the UK [52]. In addition, a recent journalistic investigation concluded that the ‘Pathways towards Lower Emissions’ report by the UN Food and Agricultural Organisation distorted research findings by Behrens and colleagues [53] by underestimating the emissions mitigation potential of reduced meat consumption, and promoting livestock intensification [54]. As a result, contradictory communications about dietary recommendations from official sources contributes to misconceptions about sustainable diets.

### Changing Communication: What should Governments be Doing?

#### Encourage Plant-based Food Consumption in Consumer-Facing Guidelines

To mitigate the harmful environmental effects of livestock production, governments should take an active role in communicating about plant-based foods [35]. Particularly, there needs to be formal recognition by governments that mainstream plant-based food consumption is necessary for a sustainable food system, while providing a range of health and economic co-benefits [55]. Additionally, in line with urgent calls for increased policy involvement to support mainstream transitions towards sustainable diets [56], food-based dietary guidelines need to be updated with recommendations for limiting meat and dairy consumption and increasing plant-based food consumption. These guidelines must be supported by key scientific evidence and accompanied by simple, actionable advice for how to meet these recommendations for both citizens and industry [33]. This may be facilitated by more formal collaborations between researchers, policymakers and other stakeholders, such as NGOs and IGOs, to foster knowledge dissemination and enhance messaging effectiveness. In fact, this strategy was recently adopted by the Danish government to help inform the ‘National Action Plan for Plant-based Foods’ [57]. Especially within industrialised nations, this could help set a standard for other governments to follow suit and promote plant-based foods.

#### Ensure Communications about Plant-based Foods are Consistent

It is imperative that any policy about sustainable dietary change align with other relevant policies to enable a gradual, successful and just reform of the current food system. For example, contradictory policies about promoting fruit and vegetable consumption whilst simultaneously subsidising livestock production can result in low public confidence about the government’s motivations to support sustainable lifestyles. Fostering transdisciplinary collaborations is crucial to help develop policy coherence that support systems-level transformation of the food sector. In fact, the new World Health Organisation project ‘The Dietary Patterns for Health and Sustainability’ is focused on building consensus between scientists and policymakers about how to conceptualise healthy and sustainable diets and the actions needed to promote these diets [58]. Facilitating greater knowledge exchange between relevant stakeholders may help streamline policy communications about plant-based foods and avoid contradictory policies that promote maintaining or increasing meat and dairy intake.

#### Demonstrate Plant-based Food Consumption within Public Institutions

Public sector procurement, including services within hospitals and the civil service, should integrate more plant-based offerings to increase the availability of sustainable food choices, or even make plant-based foods the default option (see: C40 Cities Climate Leadership Group; 59). This would help government officials and public figures to lead by example and adopt more plant-based food choices as a method of indirect communication, as well as modelling sustainable consumption as the publicly endorsed norm. Demonstrating high-impact, pro-environmental choices within public spaces could increase willingness among citizens to follow suit, as well as improving perceptions of credibility, trustworthiness and competence in the government [60]. This modelling strategy may be particularly effective to persuade high-earning citizens, whose consumption behaviour has a disproportionate impact [3, 4].

## Food Industry Communication about Plant-based Foods

### Current Communication: Industry Controls Communications about Plant-based Foods

Considering the lack of top-down communication from public bodies, information about plant-based foods is instead driven by commercial efforts (i.e. primarily from large scale retailers, manufacturers, restaurant chains, and other actors in the food service sector). These efforts typically promote meat-foods as more desirable than plant-based foods [61]. For example, in a study analysing the descriptions of ready meals in several UK supermarkets, those that contained meat were labelled with more words related to the taste, context and enjoyment of consumption (e.g. ‘delicious’, ‘crunchy’, ‘breakfast’) than those that did not contain any meat [62]. Plant-based ready meals were instead labelled with more words related to the ingredients, category information or health consequences of consumption (e.g. ‘meat-free’, ‘curry’, ‘light’), than meat ready meals. A recent study also found that plant-based foods were presented more in terms of health and sustainability claims, whereas animal-based foods were presented more in terms of emotional claims [63]. However, environmental, health and ingredient focused words do not motivate food choices in the same way that sensory, reward and situated words do, which disadvantages plant-based foods in commercial spaces [61].

Moreover, new findings suggest that mainstream consumers associate the term ‘plant-based’ with ultra-processed meat alternatives or dairy alternatives exclusively [64], despite plant-based foods encompassing a wide range of foodstuffs (see: 24). These products are often perceived as nutritionally inadequate, expensive and unenjoyable [65, 66], as part of the wider narrative around the harmful health effects of ultra-processed foods [67, 68]. Hence, this narrow conceptualisation of plant-based foods, driven by industry messaging, may deter mainstream consumers from reducing their meat and dairy consumption. Indeed, a recent study found that omnivores expected meat alternatives to be less satisfying, tasty and filling than conventional meat products [69]. However, meat and dairy alternatives are not associated with the increased risk of multiple cancers and cardiometabolic diseases that ultra-processed meat products are [70; see also: 71]. Furthermore, although meat alternatives are typically more expensive than their meat equivalents [72], research from 150 countries suggests that adopting a vegan, vegetarian or flexitarian diet in general could cut food costs by one third [73–75].

### Contextualising Communication: Industry Communications Maintain Meat-Centric Norms

In commercial settings, meat and dairy alternatives are the most frequently marketed, visible and available plant-based options, which perpetuates current norms around animal-product consumption. Framing meat and dairy alternatives as direct replacements to their animal product equivalents models traditional meals where meat or dairy is the central component [see: 76, 77]. By promoting the ideal that plant-based foods should mimic meat or dairy products, the food industry can continue a ‘business-as-usual’ approach with the types of food that are easily accessible in food purchasing environments, whilst profiting from an emerging market of sustainability-conscious consumers. However, this sets expectations that plant-based foods should be near identical to meat and dairy products with regard to taste, texture, and convenience of preparation [78].

Consequently, current strategies to promote plant-based foods commercially, such as Burger King’s exclusively plant-based restaurants in London and Vienna [79], do not align with a more comprehensive transformation of mainstream diets. As Bryant [80] suggests, meat and dairy alternatives are useful for habitual omnivores to facilitate the transition away from animal products (see also: [81] ). Nonetheless, a recent survey suggests that people have stronger intentions to substitute meat with legumes (57%) and legume-based foods (43%) rather than meat alternatives (39%; [82]). From this, it is essential to transform marketing narratives towards the idea that plant-based foods encompass a diverse range of non-animal-based foods, such as nuts, legumes, vegetables and wholegrains, that are not merely like-for-like meat or dairy substitutes.

### Changing Communication: What Should Industry Be Doing??

#### Emphasise the Taste, Reward and Enjoyment of Plant-Based Foods

Plant-based foods should be advertised as tasty, rewarding and filling to emphasise immediate food enjoyment [61]. Considering the strongest motivations for food choices are taste, price and convenience [83–85], promoting plant-based foods as attractive, affordable and accessible can increase purchasing, and thus exposure and familiarity with plant-based foods. Indeed, Turnwald & Crum [86] found that using taste-focused labels for plant-based wholefoods increased purchasing by 38% in comparison to health-focused labels, and sustained purchasing over a 2-month period. Although recent trends show that plant-based foods are starting to be labelled more in terms of their hedonic properties [63], these reward labels are typically included alongside a higher proportion of health or sustainability labels than for meat or dairy foods [61]. Furthermore, emphasising the short-term health benefits of plant-based options (e.g. feeling energised after a meal), rather than the long-term health benefits (e.g. lower risk of cardio-vascular disease) or sustainability benefits (e.g. lower carbon emissions), may be more effective for motivating momentary sustainable food choices (see: [87]).

#### Increase Communication about Plant-Based Whole Foods

Industry communication needs to highlight the diversity of plant-based foods (e.g. vegetables, pulses, legumes), and not just meat and dairy alternatives. Considering the negative connotations associated with the terms ‘vegan’ and ‘plant-based’ [88, 89], industry should focus on developing products that emphasise plant-based wholefoods as the central element of a dish (e.g. ‘lentil lasagne’ vs. ‘plant-based lasagne’). Focusing on an identifiable ingredient when naming plant-based dishes is more semantically similar to the naming conventions of animal-based dishes (e.g. ‘lentil lasagne’ vs. ‘beef lasagne’) and helps clarify the contents of the dish for mainstream consumers. Nonetheless, it is important to continue offering meat and dairy alternatives as a practical choice to help certain consumer groups shift towards more sustainable diets [35]. Therefore, promoting a wider range of plant-based foods can help multiple types of consumers eat more sustainably.

#### Rethink how Plant-based Foods are Displayed

The food industry, especially supermarkets and the out-of-home food service sector, has an important role to play in supporting consumers to adopt more sustainable diets. Private organisations are in a unique position as intermediate influencers who can shape both consumer trends and government action [90]. Nevertheless, from a commercial standpoint, diversifying from the current food system that is centered around societal meat attachment [91] may appear financially risky. Therefore, encouraging industry to use evidence-based strategies that increase the appeal of plant-based offerings can improve sales and subsequently shift the food sector towards a more sustainable model. For example, the World Resources Institute (WRI) summarised over 90 behaviour change techniques that can promote more sustainable food choices and boost purchasing, such as integrating plant-based options into meat or dairy display sections to help normalise plant-based food choices [92]. Organisations can incorporate new promotional strategies for a variety of plant-based offerings to gain revenue from a growing market of sustainability-conscious consumers. Indeed, recent efforts from large organisations such as Lidl’s Healthy and Sustainable Diets Policy and Good Food Plan [93] and Penny’s true cost campaign [94] demonstrate how the food industry can support sustainable food system transformations by prioritising affordable, accessible and available plant-based products.

## Citizen Communication about Plant-based Foods

### Current Communication: Citizens Echo Industry Communications about Plant-based Foods

Citizen communication about plant-based foods often reflects messaging that citizens are frequently exposed to in food purchase environments (see: 96). This creates a cycle between citizens and the food industry that positions habitual animal product consumption as the status-quo and plant-based foods as the alternative [62, 96], often for stereotyped “others” [97]. This is demonstrated in online discourse about food on social media, whereby user posts about meat foods included between 11 and 17% more hashtags containing features about the rewarding aspects of eating (e.g. ‘delicious’, ‘smooth’, ‘satisfying’) than posts about plant-based foods [95]. In contrast, plant-based foods were presented more in terms of their health and environmental benefits (e.g. ‘high protein’, ‘healthy’, ‘environmentally friendly’), akin to commercial settings.

Additionally, citizens also display ambiguity about what the term ‘plant-based’ refers to. In a recent qualitative study, omnivore participants displayed difficulty when describing plant-based foods, and in some cases, identified vegetarian foods as plant-based [64]. With varying definitions amongst scholars [98] regarding whether a plant-based diet is synonymous with either a flexitarian diet (i.e. including meat and dairy products) or a vegan diet (i.e. excluding all animal products), and considering new, interchangeable terms for ‘plant-based’ emerging such as ‘plant-rich’, ‘plant-focused’ and ‘plant-forward’ [99], it is no surprise that there is confusion amongst the general public [100]. This could potentially result in discouraging citizens from certain plant-based products or dishes, due to a lack of knowledge about the content of these foods.

### Contextualising Communication: Citizens Hold Established Attitudes about Plant-based Foods

The pattern of communication about plant-based foods among citizens who have the capacity to change their diets (i.e. middle-income and high-income individuals from industrialised nations; [101]) is influenced by entrenched social norms that perpetuate meat-consumption as the appropriate dietary behaviour. For example, dietary identity polarisation between omnivores and vegans drives mainstream perspectives that plant-based foods are an abnormal choice made by moral do-gooders who exclusively eat plant-based foods [102, 103]. Generational, socio-economic and gender differences also contribute to this discourse, whereby plant-based food choices are seen as a trend for younger, middle-class women [104–106]. As a result of this discourse, citizens display resistance to conceptualising a ‘sustainable’ diet as one that includes regular consumption of plant-based foods [107]. Instead, beliefs such as the ‘local is best’ fallacy (i.e. eating locally-sourced foods is the most sustainable dietary behaviour; [11,108]) provides a more convincing strategy to citizens that is less effortful than uprooting one’s meat and dairy consumption habits. This leads to mainstream consumers having widely differing ideas about what a healthy, sustainable diet looks like [83].

From this, efforts to promote plant-based foods via communication alone are unlikely to change dietary behaviour. The COM-B model posits that behaviour is shaped by one’s capability, opportunity, and motivation, with motivation being influenced, among other factors, by communication from public authorities, friends, or commercial actors [109]. Furthermore, the Grounded Cognition Theory of Desire offers insights into how communication can affect behaviour, by continuously shaping and updating cognitive representations of foods, that in turn shape behaviour [87, 110]. However, as these behavioural science theories also suggest, communication alone does not determine food choice. How citizens process information within food-related communication depends on multiple conditions, including the level of exposure, attentional capacity, prior knowledge and trust, as well as numerous motivational and situational factors [111]. For example, the success of plant-based milk acceptance is not solely attributed to positive word-of-mouth [112], but to multiple factors that facilitate behaviour change [113], including persuasive advertising (capability), rewarding consumption experiences (motivation), and widespread availability (opportunity).

### Changing Communication: What Should Citizens be Doing??

#### Talk about Plant-based Foods in More Appealing Ways

Citizens can improve public discourse about sustainable diets by describing, presenting and categorising plant-based foods in more appealing ways. Research has found that vegans think about plant-based foods in terms of consumption and reward, but do not convey these attitudes when talking about plant-based foods to others [114]. Instead, vegans tend to emphasise the health, environmental or socio-political features of plant-based foods in public communications, which may inflate dietary polarisation with omnivores [115]. By demonstrating to omnivores that vegans aren’t ‘taste martyrs’ but enjoy the foods they eat frequently just as much as omnivores do, emphasising the satisfying aspects of plant-based foods can help bridge the gap between these polarised groups [116]. Furthermore, professionals who work in the food sector may need to consider whether their own attitudes about plant-based foods may impact their industry outputs. There is an opportunity for these groups to influence communications about plant-based foods towards the hedonic properties of sustainable food choices.

#### Recognise Eating Sustainably is Not an All or Nothing Approach

People find smaller steps towards meat reduction more acceptable than radical dietary change. For instance, smaller meat portion sizes are directly attributed to decreasing meat consumption in the UK [117]. Transitioning to low-meat meals is likely easier than meat-free meals, as it presents fewer barriers related to taste preferences, cooking skills, and perceived social norms [78]. Additionally, in an intervention study testing meat-reduction strategies, the two most popular actions were ‘*make at least one of your main meals vegetarian*’ and ‘*double the veg*,* halve the meat*’, while ‘*go plant-based for the whole day*’ was the least chosen action [118]. This is also reflected in meat and dairy reducers’ accounts of successful dietary change [81]. Therefore, it is important to emphasise in public discourse that sustainable diets are flexible, and can include a small amount of animal products (e.g. EAT-Lancet Diet; [17]), rather than solely plant-based foods. Ultimately, making gradual plant-based food choices, such as one additional plant-based meal or plant-based day per week, may help citizens switch faster to sustainable eating behaviours than immediately shifting to a fully plant-based diet.

#### Use Plant-based Food as a Starting Point to Talk about Climate Change

The topic of sustainable diets can be an accessible starting point for discussing climate change more generally. Around 75% of citizens in the UK are concerned about climate change [119], but many people find it difficult to express their concerns [120]. Talking about climate change can unlock information sharing, social support, and opportunities for collective action [121], especially among citizens with the agency and capacity to participate. In addition, the success of recent citizen assemblies (e.g. France, Ireland), including food-focused citizen assemblies (e.g. Sweden), show that there is strong public support for sustainability-focused policies when citizens are given time, space and resources for discussions about the climate crisis [122–125]. Thus, communicating about positive climate action can empower citizens to help shift conceptualisations of climate change from issue-based to action-based [126]. From this perspective, talking about sustainable diets can help open discussions about interconnected or overarching issues, such as other carbon-intensive behaviours (e.g. flying), individual responsibility versus structural change, and high-emitting lifestyles of the richest consumers [101]. Such discussions can result in greater citizen advocacy for immediate climate action, which can subsequently influence policymakers and industry to follow suit [127].

**Fig. 1:**
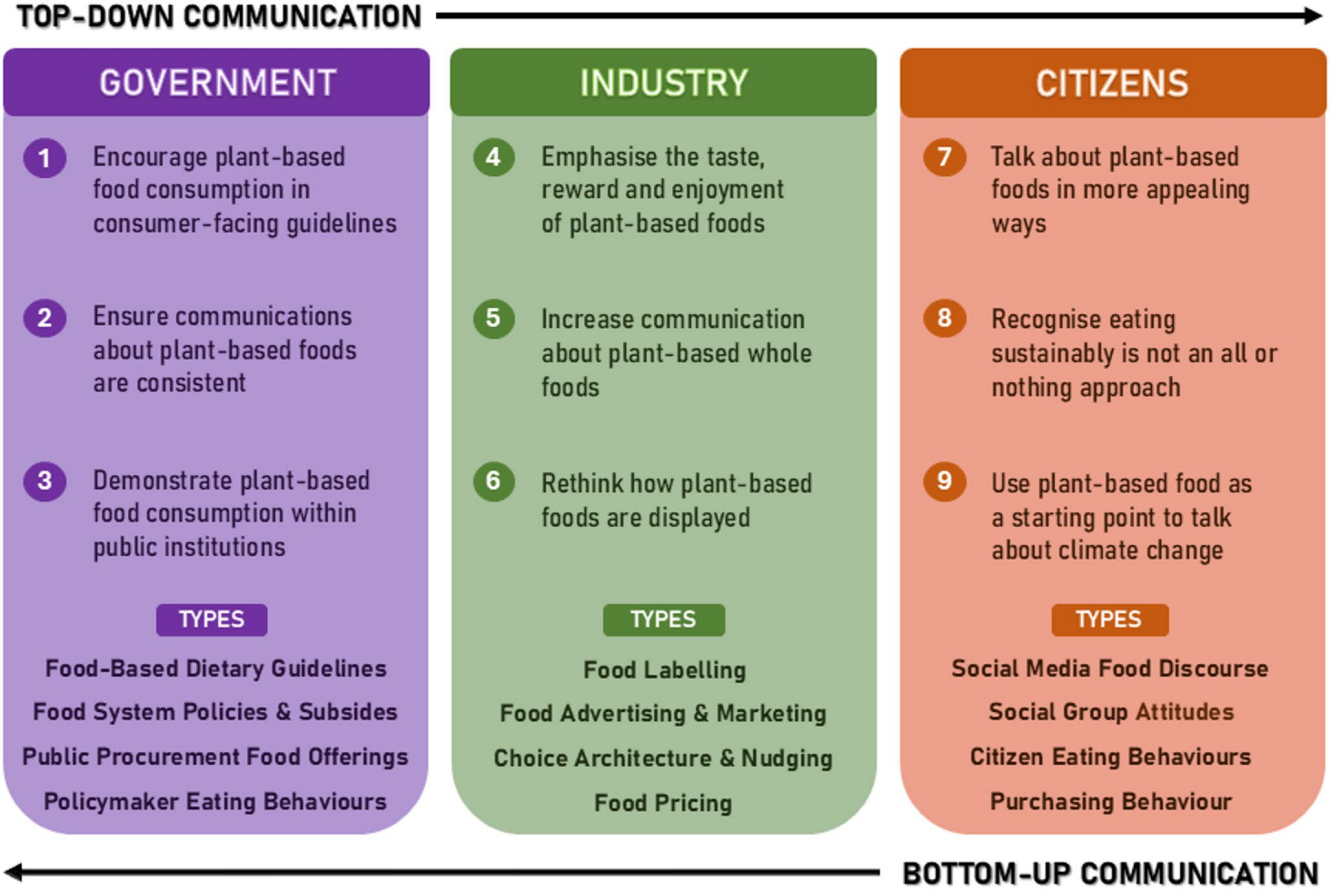
9 Recommendations for Government, Industry and Citizens

## Conclusions

In this paper, we discussed how the government, food industry and citizens communicate about plant-based foods in the context of the climate crisis. Overall, this communication tends to be unclear, contradictory and unsupportive of mainstream transitions towards more sustainable diets. The lack of communication from governments about plant-based foods has left the food industry to define and market these products, which has reinforced the perception that they are merely substitutes for meat and dairy. Citizens mirror this in their own discourse, creating a negative feedback cycle of commercially driven consumer demand that prioritises meat and dairy foods and keeps plant-based foods in the ‘alternative’ space.

To support the transition to sustainable food systems, communication about plant-based foods and sustainable diets should be unified and consistent across multiple actors in the food system to normalise plant-based food choices. We have made realistic yet impactful recommendations (see Fig. 1), which together may create a food environment that is more conducive to sustainable food choices. For example, by integrating multiple policies to help facilitate a just transition towards sustainable diets (Recommendation 2), the food industry is encouraged to promote a wider variety of plant-based products (Recommendation 5), which can then positively impact perceptions of plant-based foods among citizens (Recommendation 7).

In sum, stakeholders from across the food sector can use effective communication to change behaviours and beliefs in this complex system, and ultimately contribute to changing the food system’s goals [see: 128] to maximise benefits for human and planetary health. Nonetheless, the way we talk about foods is just one part of shifting the narrative around sustainable eating. Critically, communications about plant-based foods must be adopted in line with broader systemic changes to support sustainable food system transformation.

## Data Availability

No datasets were generated or analysed during the current study.
